# Effect of Home Enteral Nutrition on Nutritional Status, Body Composition and Quality of Life in Patients With Malnourished Intestinal Failure

**DOI:** 10.3389/fnut.2021.643907

**Published:** 2021-07-01

**Authors:** Xuejin Gao, Yupeng Zhang, Li Zhang, Sitong Liu, Hao Liu, Da Zhou, Jieshou Li, Xinying Wang

**Affiliations:** ^1^Affiliated Jinling Hospital, Research Institute of General Surgery, Medical School of Nanjing University, Nanjing, China; ^2^Affiliated Jinling Hospital, Research Institute of General Surgery, Southern Medical University, Guangzhou, China

**Keywords:** intestinal failure, home enteral nutrition, quality of life, nutritional status, phase angle

## Abstract

**Background:** The ultimate goal of intestinal failure (IF) management is to maintain optimal nutritional status, improve the quality of life (QoL), and promote intestinal adaptation. Enteral nutrition support is safe and effective in patients with IF and plays a central role in the management of patients with IF. The purpose of this study was to evaluate the effect of home enteral nutrition on nutritional status, body composition (BC), QoL and other clinical outcomes in malnourished patients with intestinal failure.

**Methods:** This prospective observational study included 166 malnourished patients with intestinal failure presented to Jinling Hospital from January 2016 to October 2018. All patients were supported with home enteral nutrition after discharge. We evaluated clinical outcomes, including nutritional status, BC, phase angle (PhA), QoL, mortality, gastrointestinal complications related to enteral feeding, and other clinical outcomes at 1, 3, and 6 months after discharge.

**Results:** Body weight, BC, and other nutritional parameters were maintained or significantly increased during the period of home enteral nutrition after discharge (*p* < 0.01). Especially, the quality of skeletal muscle mass in body composition was significantly improved (*p* < 0.01). SF-36 quality of life scores was significantly improved (discharged at 6 months: reported health transition 40.7 ± 12.1 vs. 69.3 ± 16.3, *p* < 0.01). There were no differences between hospital and out of hospital with respect to tube-related or gastrointestinal complications. Advanced age, disease type, and poor nutritional status were risk factors for poor clinical outcomes.

**Conclusions:** Home enteral nutrition support is effective for malnourished patients with intestinal failure. It improves nutritional status, BC, PhA, and QoL.

**Clinical Trial Registration:** identifier: ChiCTR2000035145.

## Introduction

Intestinal failure (IF), a rare type of organ failure, is defined as “the reduction of gut function below the minimum necessary for the absorption of macronutrients and/or water and electrolytes, such that intravenous supplementation (IVS) is required to maintain health and/or growth” (1). Based on the onset, metabolic and expected outcome criteria, the functional classification of IF including Type I (acute, short-term, and usually self-limiting condition), Type II (prolonged acute condition), and Type III (chronic condition) (2).

The goal of IF management is to maintain optimal nutritional status, reduce complications, improve quality of life, and promote intestinal adaptation or enteral autonomy (2–4). In the case of patients with intestinal dysmotility or an intestinal mechanical obstruction mainly depend on parenteral nutrition to sustain life, due to insufficient intestinal nutrition. Although, PN is a life-saving therapy for patients with IF (5, 6). Long-term use of PN is associated with many complications (5), including PN-associated liver disease, catheter-related infections, thrombosis and metabolic complications (5–8). At present, it is accepted that enteral nutrition (EN) can enhance the intestinal adaptation in patients with IF (9). Therefore, EN should be started as soon as possible the gut is functional. Compared with parenteral nutrition, enteral nutrition as a preferred option has the advantages of being more economical, more convenient, and safer. Furthermore, enteral feeding conforms to physiological functions, which could protect the gastrointestinal barrier, immune function, and motility (10). Therefore, it is important for IF patients to successfully implement EN feeding to significantly improve the intestinal rehabilitation process and the patient's QoL (5).

Home enteral nutrition (HEN) therapy delivers nutrients and/or fluids to the gastrointestinal tract (GI) through tube or stoma to patients who are medically stable and unable to meet oral nutrition (11). Since its introduction in the 1970's, HEN has been identified as a reliable and effective nutritional intervention (12). Enteral nutrition is started during a hospital stay and continued as a long-term home enteral nutrition therapy (12), which is only minor different from the indication for hospital enteral nutrition.

Nutritional support treatment is usually indicated in patients who are malnourished or at high risk of malnutrition in the hospital. When the patients discharged from the hospital, HEN can be used as a supplementary life-sustaining therapy (12) and can be maintained or even saved the lives patients who unable to meet energy needs via daily oral intake (13, 14). Home enteral nutrition improves prognosis in patients with severe chronic diseases and allows the integration of patients with their families and society (15), thereby improving QoL (12). Moreover, it is associated with improved health outcomes, lower readmission rates, and reduced medical costs (16, 17). EN is safe and effective for patients with IF (18) and plays a central role in the management of patients with intestinal failure (3). For these reasons, as a cost-effective and reliable complementary treatment method, home enteral nutrition is also quite essential for patients with IF who need nutritional support after discharged. HEN combined with home parenteral nutrition can prevent further deterioration of the nutritional status in malnourished patients with IF. However, large sample prospective studies specifically aimed at investigating the effects of home enteral nutrition in malnourished patients with IF are lacking.

Therefore, the main purpose of this study was to determine the effect of HEN on nutritional status, body composition, phase angle (PhA), quality of life, and physiological function in malnourished patients with IF.

## Materials and Methods

### Study Design and Ethics Approval

The protocol for this prospective observational study conformed to the ethical guidelines of the Declaration of Helsinki and was approved by the Research Ethics Committee of the Jinling Hospital. In accordance with the Austrian law and Research Ethics Committee guidelines, all participants or their guardians obtained written informed consent. The trial was registered at Chinese Clinical Trial Registry (ChiCTR2000035145).

### Patients and Setting

The data of 166 patients who received HEN treatment in the Clinical Nutrition Treatment Center of Jinling Hospital from January 2016 to October 2018 were analyzed. Patients receive appropriate HEN treatment according to standard protocols from the nutrition support team.

The definition of intestinal failure is as described above, according to ESPEN guidelines (1). Malnutrition is defined according to the 2015 ESPEN Consensus Statement (19). Adult patients with IF were eligible if they met the following inclusion criteria: age ≥18 years; inability to meet nutritional requirements orally; estimated duration of enteral nutrition therapy at home for at least 4 weeks; stable clinical status; patient and family acceptance; and appropriate and safe home environment. Exclusion criteria included contraindication for enteral nutrition; life expectancy of fewer than 2 months; pregnancy; severe renal or liver dysfunction; participation in another clinical study; and inability or unwillingness to provide informed consent.

Standardized enteral nutrition was provided according to a nutritional protocol. The energy requirement target was calculated based on actual body weight as 25–30 kcal/kg, and protein requirements ranged from 1.0 to 1.5 g/kg/day. Before being discharged from the hospital, members of the nutrition team will train caregivers who would assist them at home (family members or informal caregivers) to implement HEN therapy (20). The specific training content mainly includes the safe utilization of the infusion pump, proper use and storage of enteral feeding formulas, the management of drugs and water through enteral feeding devices, and the protection of tube position (21). Nutrition support team members followed up on patients once a month. The home visiting staff performed physical examinations and assessments of each patient following standard procedures. During each home visit, assessments included body weight, body composition, blood pressure, blood glucose, and QoL using the SF-36 Health Survey.

In the standard protocol, we define the conditions and cut-off values for the diagnosis of HEN treatment complications. Gastrointestinal complications (GIC) were defined as follows: constipation: despite the use of constipation drugs, no bowel movement for more than 3 days; abdominal distension: abdominal changes during the physical examination with tympany and/or no bowel sounds; diarrhea: loose and watery bowel movements (stools), with more than three stools per day; vomiting: enteral formula ejected through the mouth; and aspiration: diet presence in the airway or respiratory tract (with or without exteriorization) (21, 22). Metabolic complications were defined as follows: hyperglycemia (>11.1 mmol/L) and hypoglycemia (<4.4 mmol/L); hypernatremia (>150 mmol/L); hyponatremia (<136 mmol/L); hyperkalemia (>5.5 mmol/L); hypokalemia (<3.5 mmol/L); high blood urea nitrogen levels (>405 mg/dl); and dehydration based on clinical signs, such as oliguria (<400 mL urine/day) (21). The hospital physician specialized in clinical nutrition undertook the prevention and treatment of GIC and metabolic complications, high blood urea nitrogen levels, and dehydration after notification by the home visiting staff (21).

When tube-related complications occurred, including displacement, occlusion and breakage, infection around the wound exit site, and the presence of granulation tissue, home visiting staff diagnosed them and when possible addressed them directly at the patients' homes (21).

#### Bioelectrical Impedance Analysis

In Body S10 (InBody Co, Ltd., Seoul, South Korea) was selected to perform BIA measurement to assess body composition. The device has a tetrapolar eight-point contact electrode system that contains six different Frequency (1, 5, 50, 250, 500, and 1,000 kHz) (23). The patient is required to fasting and keep the quiescent condition for 3 h in advance, and then take a supine position during the measurement, with both hands placed on both sides of the body's midline (23). PhA derived from BIA was determined as follows: PhA = arctangent (reactance/resistance)^*^(180/π). Reactance and resistance were measured at 50 kHz (24).

### Laboratory Analysis

Blood samples were drawn in the morning after overnight fasting. Hemoglobin, serum levels of albumin, prealbumin, transferrin, retinol binding protein, fibronectin, insulin-like growth factor 1(IGF-1), liver enzymes and bilirubin, electrolytes, urea nitrogen, and creatinine were measured using routine methods at the Department of Laboratory Medicine, Jinling Hospital or the local hospital laboratory.

### Data Collection

All participants of baseline data collection are starting home enteral nutrition treatment, including demographic data (age, gender, primary disease, etc.), information about nutrition therapy, nutritional status, body composition, PhA, and QoL. The implementation of enteral feeding relies on infusion pump or gravity infusion.

Information on the frequencies and types of complications associated with HEN therapy, nutritional status, body composition, PhA, quality of life, and the clinical outcomes of the HEN therapy (death, moving from tube-feeding to oral feeding, and work status) was collected during hospitalization and at 1, 3, and 6 months after discharge.

### Data Analyses

Patient characteristics are displayed by descriptive statistics, and then statistical methods are selected according to the data type and distribution status. Continuous variables are expressed as mean ± SD or median and range, and categorical variables are expressed as absolute values and relative frequencies. The incidence rates of complications were compared for inpatients and outpatients using the chi-square test. Nutritional status, body composition, PhA, and QoL were analyzed using one-way ANOVA. Logistic regression analysis was applied to assess which risk factors were correlated with survival. All statistical analysis was using SPSS 21.0 software (Statistical Program for Social Sciences, SPSS Inc, Chicago, IL, USA) and a value of *P* < 0.05 was considered statistically significant.

## Results

**Figure 1:**
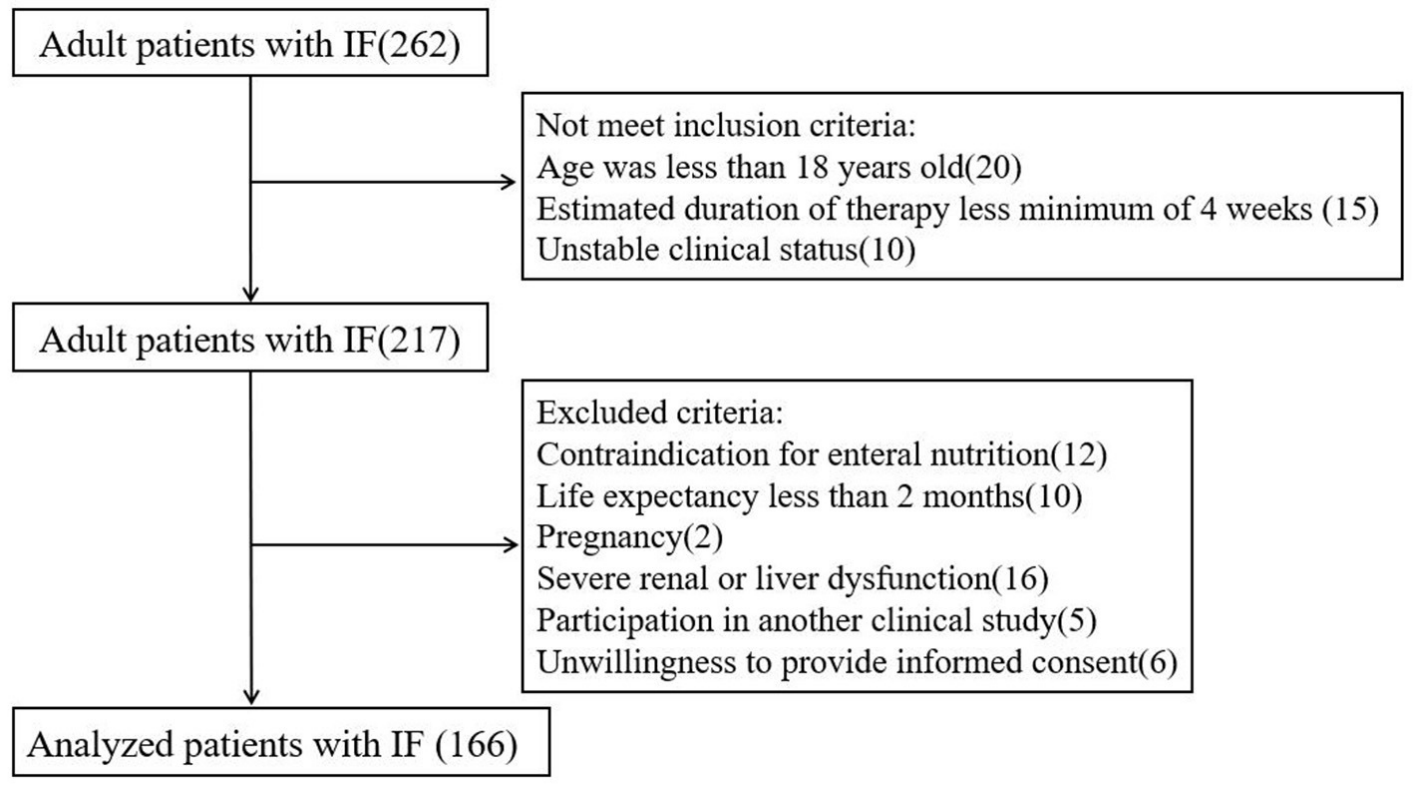
The flow chart of study design and patient selection.

A total of 262 patients were admitted to our clinical nutrition center between January 2016 and October 2018. According to the inclusion and exclusion criteria, 166 patients were finally included in the analysis (Figure 1).

### Patient Population

In total, 166 patients (57.8% female, 42.2% male; mean age 46.2 ± 16.5 years) were enrolled. The main reasons for requiring HEN were short bowel syndrome (SBS) (25.9%), extensive small bowel mucosal disease (21.1%), intestinal dysmotility (19.3%), mechanical obstruction (15.1%), intestinal fistula (2.4%), and others (16.3%). The functional classification of IF was as follow: Type II (14.5%) and Type III (85.5%). Baseline characteristics are displayed in Table 1.

**Table 1:** Patients and disease characteristics at baseline.

**Variable**	**Range**
Total no. of patients	166
Age (years)	46.2 ± 16.5
Male/female	70/96
Height (cm)	165.8 ± 7.8
Weight (kg)	49.4 ± 10.8
BMI kg/m^2^	17.9 ± 3.4
Duration after diagnosis of IF (months), median (IQR)	3 (1.5–22)
**Functional classification**	
Type **II** (prolonged acute)	24 (14.5)
Type **III** (chronic)	142 (85.5)
**Pathophysiological classification**	
Short bowel syndrome	43 (25.9)
Mesenteric infarction (arterial or venous thrombosis)	20
Crohn's disease	8
Radiation enteritis	6
Surgical complications	5
Intestinal volvulus	2
Abdominal trauma	2
Extensive small bowel mucosal disease	35 (21.1)
Crohn's disease	10
Radiation enteritis	12
Chemotherapy related enteritis	9
Celiac disease	4
Intestinal dysmotility	32 (19.3)
Post-operative	15
Systemic inflammatory	9
Chronic intestinal pseudo-obstruction	8
Mechanical obstruction	25 (15.1)
Obturation (polypoid tumors, feces)	11
Intrinsic bowel lesions (neoplastic, IBD, anastomotic)	8
Extrinsic lesions (abdominal adhesions, neoplasia, volvulus)	6
Intestinal fistula	4 (2.4)
Radiation enteritis	2
Neoplastic	1
Trauma	1
Others	27 (16.3)
Miscellaneous	25
Congenital diseases	2

*Data are number of participants (%) or mean (SD) unless otherwise noted*.

*BMI, body mass index*.

The majority of patients were fed by naso-intestinal tube (37.3%), nasogastric tube (42.2%), and percutaneous endoscopic gastrostomy/jejunostomy (PEG-J) (6.6%), and most infusions were pump infusion (83.1%) rather than bolus (9.6%). The mean duration of home enteral feeding (HEF) was within 3 months in 66.3% and 6 months in 33.7%. The main types of enteral nutrition products are standard type (Table 2).

**Table 2:** Techniques used for HEN.

**GI tract access**	
PEG-J	11 (6.6)
Nasogastric tube	70 (42.2)
Naso-intestinal tube	62 (37.3)
Jejunostomy	7 (4.2)
Oral administration	16 (9.6)
**Mean duration of HEN (months) % of patients treated:**	
<1 months	23 (13.9)
1–3 months	87 (52.4)
3–6 months	56 (33.7)
**Infusion technique (pts, %)**	
Bolus technique	16 (9.6)
Gravity set	12 (7.2)
Pump infusion	138 (83.1)
**Person responsible for HEN**	
Patient him/herself	92 (55.4)
Caregiver	74 (44.6)
**Enteral diet used (%):**	
Standard	113 (68.1)
Fiber-rich	5 (3.0)
Protein-rich	6 (3.6)
Energy-dense	2 (1.2)
Diabetic	6 (3.6)
Oligopeptide	25 (15.1)
Blenderized diet	9 (5.4)

*Data are number of participants (%)*.

### Body Weight, Body Composition, and Phase Angle

Compared with the values before admission, the body weights, body composition, and PhA significantly increased during home enteral nutrition, especially within 3 months after discharge (*p* < 0.05) (Table 3). BIA analysis was also indicated that the skeletal muscle mass, soft lean mass, and fat-free mass of body composition significantly improved (discharged at 6 months: skeletal muscle 21.81 ± 5.24 vs. 22.90 ± 5.69, *p* < 0.05; soft lean mass 38.41 ± 8.16 vs. 40.38 ± 9.19, *p* < 0.01; fat-free mass 41.01 ± 8.58 vs. 43.13 ± 9.72, *p* < 0.01) (Table 3).

**Table 3:** Body weight, body composition, and phase angle change.

**Variable**	**Pre-hospital**	**First month after hospital discharge**	**Three months after hospital discharge**	**Six months after hospital discharge**	***p*-value**
Weight (kg)	49.4 ± 10.8	55.5 ± 12.6	54.4 ± 12.9	53.8 ± 13.3	<0.001^a^^,^^b^^,^^c^
ICW (L)	18.26 ± 4.02	20.10 ± 4.51	18.99 ± 4.51	19.09 ± 4.36	0.0020^a^^,^^d^^,^^e^
ECW (L)	11.77 ± 2.42	12.63 ± 2.77	12.17 ± 2.74	12.51 ± 2.95	0.020^a^^,^^c^
TBW (L)	30.03 ± 6.33	32.72 ± 7.19	31.16 ± 7.17	31.61 ± 7.20	0.006^a^^,^^c^^,^^d^
ECW/TBW (%)	0.39 ± 0.02	0.39 ± 0.02	0.39 ± 0.02	0.40 ± 0.02	<0.001^a^^,^^d^^,^^e^^,^^f^
TBW/FFM (%)	73.18 ± 0.57	73.06 ± 0.63	73.12 ± 0.51	73.26 ± 0.60	0.018^e^^,^^f^
Protein (Kg)	7.89 ± 1.74	8.69 ± 1.95	8.20 ± 1.95	8.26 ± 1.90	0.002^a^^,^^d^^,^^e^
Mineral (Kg)	3.10 ± 0.59	3.38 ± 0.76	3.25 ± 0.72	3.27 ± 0.71	0.004^a^^,^^b^^,^^c^
FAT (Kg)	8.35 ± 5.38	10.71 ± 6.73	11.82 ± 6.44	10.62 ± 7.81	<0.001^a^^,^^b^^,^^c^
SLM (Kg)	38.41 ± 8.16	41.95 ± 9.25	39.88 ± 9.22	40.38 ± 9.19	0.004^a^^,^^d^
FFM (Kg)	41.01 ± 8.58	44.79 ± 9.83	42.62 ± 9.78	43.13 ± 9.72	0.004^a^^,^^b^^,^^d^
SMM (Kg)	21.81 ± 5.24	24.21 ± 5.88	22.75 ± 5.88	22.90 ± 5.69	0.002^a^^,^^d^^,^^e^
PBF (%)	16.34 ± 8.51	18.63 ± 9.66	21.00 ± 8.95	18.42 ± 10.99	<0.001^c^
BCM (Kg)	26.15 ± 5.75	28.79 ± 6.46	27.19 ± 6.45	27.35 ± 6.24	0.002^a^^,^^d^^,^^e^
BMC (Kg)	2.61 ± 0.50	2.84 ± 0.65	2.73 ± 0.61	2.75 ± 0.60	0.004^a^^,^^b^^,^^c^
AC (cm)	22.86 ± 3.43	24.94 ± 4.44	24.36 ± 3.50	24.37 ± 4.95	<0.001^a^^,^^b^^,^^c^
AMC (cm)	19.28 ± 2.79	20.52 ± 3.29	19.97 ± 2.51	20.22 ± 4.09	0.004^a^^,^^c^
Waist Cir. (cm)	63.26 ± 7.18	65.41 ± 7.66	66.68 ± 8.68	66.07 ± 9.86	0.001^b^^,^^c^
VFA (cm^2^)	37.47 ± 22.22	41.02 ± 28.06	49.44 ± 30.92	49.29 ± 38.04	<0.001^b^^,^^c^
RA phase angle	4.57 ± 1.83	5.12 ± 2.20	4.59 ± 1.05	4.48 ± 1.39	0.002^a^^,^^d^^,^^e^
LA phase angle	4.38 ± 1.70	4.90 ± 2.46	4.41 ± 1.07	4.70 ± 3.57	0.137
TR phase angle	5.74 ± 3.36	6.00 ± 2.64	4.73 ± 1.92	5.61 ± 4.27	0.002^b^^,^^d^
RL phase angle	4.89 ± 1.86	5.84 ± 2.08	5.21 ± 1.89	4.72 ± 1.74	<0.001^a^^,^^d^^,^^e^^,^^f^
LL phase angle	4.84 ± 1.96	5.83 ± 2.05	5.13 ± 1.80	4.71 ± 1.82	<0.001^a^^,^^d^^,^^e^
Average phase angle	4.88 ± 1.85	5.54 ± 1.72	4.81 ± 1.39	4.84 ± 2.01	<0.001^a^^,^^d^^,^^e^

*Data are mean (SD) unless otherwise noted. a, b, c, d, e, f indicates pair-wise differences were significant at p < 0.05. Where*

a *indicates p < 0.05 for pre-hospital vs. first month after hospital discharge,*

b *indicates p < 0.05 for pre-hospital vs. three months after hospital discharge,*

c *indicates p < 0.05 for pre-hospital vs. 6 months after hospital discharge,*

d *indicates p < 0.05 for first month after hospital discharge vs. 3 months after hospital discharge,*

e *indicates p < 0.05 for first month after hospital discharge vs. six months after hospital discharge, and*

f *indicates p < 0.05 for 3 months vs. 6 months after hospital discharge*.

*ICW, intracellular water; ECW, extracellular water; TBW, total body water; SLM, soft lean mass; FFM, fat free mass; SMM, skeletal muscle mass; PBF, percent body fat; BCM, body cell mass; BMC, bone mineral content; AC, arm circumference; AMC, arm muscle circumference; Waist Cir., waist circumference; VFA, visceral fat area; RA, right arm; LA, left arm; TR, trunk; RL, right leg; LL, left leg; PhA, phase angle*.

### Other Nutritional Parameters

At 1, 3, and 6 months after discharge, the serum concentrations of biochemical nutritional indicators (albumin, prealbumin, retinol binding protein, transferrin, fibronectin, and IGF-1) were significantly higher in HEN patients, compared with the pre-hospital values (*p* < 0.01) (Table 4).

**Table 4:** Other nutritional parameters.

**Variable**	**Pre-hospital**	**First month after hospital discharge**	**Three months after hospital discharge**	**Six months after hospital discharge**	***p*-value**
Albumin (g/L)	36.1 ± 6.6	37.1 ± 5.4	41.2 ± 4.0	39.9 ± 4.6	<0.001^b^^,^^c^^,^^d^^,^^e^
Prealbumin (mg/L)	159.5 ± 73.7	166.1 ± 62.0	182.6 ± 81.4	198.3 ± 71.8	<0.001^c^^,^^e^
Retinol binding Protein (mg/L)	31.4 ± 14.7	31.3 ± 14.7	35.1 ± 10.3	39.3 ± 19.3	<0.001^c^^,^^e^
Transferrin (g/L)	2.23 ± 0.67	2.43 ± 0.64	2.44 ± 0.78	2.42 ± 0.70	0.0230^a^
Fibronectin (mg/L)	192.3 ± 54.3	210.1 ± 33.0	211.0 ± 59.9	217.2 ± 39.7	0.0010^a^^,^^c^
IGF-1 (ug/L)	104.5 ± 86.8	139.6 ± 69.3	191.6 ± 112.9	170.5 ± 127.7	<0.001^a^^,^^b^^,^^c^^,^^d^

*Data are mean (SD) unless otherwise noted. a, b, c, d, e, f indicates pair-wise differences were significant at p < 0.05. Where*

a *indicates p < 0.05 for pre-hospital vs. first month after hospital discharge,*

b *indicates p < 0.05 for pre-hospital vs. 3 months after hospital discharge,*

c *indicates p < 0.05 for pre-hospital vs. 6 months after hospital discharge,*

d *indicates p < 0.05 for first month after hospital discharge vs. 3 months after hospital discharge,*

e *indicates p < 0.05 for first month after hospital discharge vs. 6 months after hospital discharge and*

f *indicates p < 0.05 for 3 months vs. 6 months after hospital discharge*.

*IGF-1, insulin-like growth factor-1*.

### Quality of Life

Overall, the reported health transition scores were improved at all three time points, with respect to baseline values (40.7 ± 12.1 vs. 57.4 ± 15.6 vs. 61.7 ± 17.6 vs. 69.3 ± 16.3, *p* < 0.001). The self-completed questionnaire includes 36 items, divided into eight domains (physical functioning [PF], role-physical [RP], bodily pain [BP], general health [GH], vitality [VT], social functioning [SF], role-emotional [RE], and mental health [MH]) scores, which also significantly improved during home enteral nutrition after discharge (*p* < 0.001) (Table 5).

**Table 5:** HEN quality of life scores.

**Variable**	**Pre-hospital**	**First month after hospital discharge**	**Three months after hospital discharge**	**Six months after hospital discharge**	***p*-value**
RHT	40.7 ± 12.1	57.4 ± 15.6	61.7 ± 17.6	69.3 ± 16.3	<0.001^a^^,^^b^^,^^c^^,^^e^^,^^f^
PF	45.4 ± 12.0	59.2 ± 11.5	60.9 ± 15.0	62.9 ± 16.7	<0.001^a^^,^^b^^,^^c^
RP	36.4 ± 12.5	55.6 ± 16.6	58.7 ± 19.2	59.0 ± 19.6	<0.001^a^^,^^b^^,^^c^
BP	56.2 ± 10.3	66.6 ± 10.0	71.9 ± 14.2	70.8 ± 14.3	<0.001^a^^,^^b^^,^^c^^,^^d^^,^^e^
5GH	51.7 ± 9.1	64.9 ± 10.5	67.0 ± 12.5	66.1 ± 14.4	<0.001^a^^,^^b^^,^^c^
VT	50.0 ± 9.0	63.7 ± 10.8	66.7 ± 14.2	66.8 ± 15.2	<0.001^a^^,^^b^^,^^c^
SF	56.7 ± 6.1	69.4 ± 7.6	72.1 ± 9.9	71.8 ± 10.2	<0.001^a^^,^^b^^,^^c^^,^^d^
RE	46.1 ± 16.2	71.1 ± 21.7	73.4 ± 16.0	70.5 ± 16.2	<0.001^a^^,^^b^^,^^c^
MH	42.4 ± 5.3	57.7 ± 9.1	63.8 ± 17.5	65.6 ± 18.3	<0.001^a^^,^^b^^,^^c^^,^^d^^,^^e^

*Data are mean (SD) unless otherwise noted. a, b, c, d, e, f indicates pair-wise differences were significant at p < 0.05. Where*

a *indicates p < 0.05 for pre-hospital vs. first month after hospital discharge,*

b *indicates p < 0.05 for pre-hospital vs. 3 months after hospital discharge,*

c *indicates p < 0.05 for pre-hospital vs. 6 months after hospital discharge,*

d *indicates p < 0.05 for first month after hospital discharge vs. 3 months after hospital discharge,*

e *indicates p < 0.05 for first month after hospital discharge vs. 6 months after hospital discharge, and*

f *indicates p < 0.05 for 3 months vs. 6 months after hospital discharge.*

*RHT, reported health transition; PF, physical functioning; RP, role physical; BP, bodily pain; GH, general health; VT, vitality; SF, social functioning; RE, role emotional; MH, mental health; HEN, home enteral nutrition*.

### Complications

Compared with the hospital treatment period, there were no significant increases in gastrointestinal, metabolic, or mechanical complications during HEN (*p* > 0.05) (Table 6).

**Table 6:** Complications of HEN.

**Variable**	**In hospital complications**	**Out of hospital complications**	***p*-value**
Tube complications			0.985
Reflux of feed/vomiting	4	5	
Tube displacement or migration	3	3	
Inadvertent tube removal	4	6	
Tube fracture	1	1	
Leakage around insertion site	1	2	
Tube occlusion	6	7	
Gastrointestinal complications			0.907
Gas/bloating	10	7	
Nausea/vomiting	5	6	
Diarrhea	6	7	
Constipation	8	6	
Bleeding	0	0	
Other complications			0.648
Fever	6	3	
Pain	5	6	
Aspiration	0	0	
Pneumonia	11	8	

*Data are number of participants (%). HEN, home enteral nutrition*.

### Other Clinical Outcomes

The outcomes of the HEN therapy (moving from tube-feeding to oral feeding, received calories and protein from EN and work status) significantly improved at 6 months after discharge (Supplementary Table 1). There were 18 deaths in the study population (Supplementary Table 1). The results of logistic regression analysis indicate that the risk factors for mortality in malnourished patients with IF who were treated with HEN included advanced age (age >65 years), disease type (cancer), and poor nutritional status (BMI < 16.5 kg/m^2^) (Supplementary Table 2).

## Discussion

Weight loss and the accompanying malnutrition are the major issues in patients with IF. This disease could give rise to reduced food intake, malabsorption, and increased metabolism, thereby increasing the risk of deteriorating nutritional status and ultimately negatively affecting clinical outcomes. Previous studies have shown the importance of enteral nutrition in reducing weight loss in patients with IF or intestinal insufficiency (25, 26). However, there is a lack of prospective studies evaluating the effects of long-term use of HEN in these patients.

In the present study, we assessed the effect of HEN on nutritional status, BC, PhA, and QoL in malnourished patients with IF. The results suggested that patients treated with HEN succeeded in maintaining stable body weight and body composition and PhA significantly increased during home enteral nutrition within the 1st month of discharge. Our results found that the serum concentrations of biochemical nutritional indicators (albumin, prealbumin, retinol binding protein, transferrin, fibronectin, and IGF-1) were significantly higher at 1, 3, and 6 months after discharge. Our results show that home enteral nutrition can improve nutritional status in patients with IF. We also identified changes in body composition and PhA in patients with intestinal failure in home enteral nutrition that no previous study has reported.

According to the ASPEN guidelines, BIA is a practical, portable, non-invasive body composition assessment tool (27). PhA can be used as an indicator of cell membrane quality and cell function (24). Low PhA is associated with increased number of readmissions, prolonged length of hospital stays, and deteriorated mortality in patients with intestinal failure (24). PhA may be a predictive factor for acute complications, muscle mass, and nutritional status (28). Indeed, we found that the PhA of patients with intestinal failure increased at 1 month and decreased at 3 and 6 months. These findings suggest that further researches are necessary to explore the physiologic and cellular mechanisms associated with PhA. However, due to the variability of body composition estimated within studies and the limited number of studies applying the same equipment, it was difficult to consolidate data by manufacturer to support summary statistics (27). It is urgent in future studies to cross verify the PhA values between devices of different brands or standardize their PhA values through device specific reference values, to enable comparisons of outcomes between studies applying different BIA devices (24, 29).

In the present study, we found that skeletal muscle mass, soft lean mass, and fat-free mass markedly increased with home enteral nutrition. It is well-known that as a highly plastic tissue, skeletal muscle is indispensable in human health and disease (30). Disease-related malnutrition is mainly manifested as decreased muscle mass and function, and this phenotype is related to loss of independence and decreased quality of life (30). Therefore, maintaining the quality of skeletal muscle is essential to achieve a normal nutritional status. The GLIM consensus has regarded skeletal muscle quality as the decisive criterion for diagnosing malnutrition due to its role in responding to trauma and disease (31, 32). This maintenance of body weight, body composition (skeletal muscle mass), PhA, and other nutritional indicators can be attributed to the increased caloric and protein intake from home enteral nutrition. The caloric intake was significantly higher in patients supported with HEN compared with baseline. Our results found a positive effect of home enteral nutrition on the up-regulation of IGF-I concentration. Previous research findings that IGF-1 contributes to enhance muscle function by increasing the production of muscle satellite cells and promoting the synthesis of muscle contractile proteins and mediator of muscle growth and repair (33, 34).

Compared with baseline values, the SF-36 scores, including the reported health transition, was significantly increased at 1, 3, and 6 months after discharge, suggesting that HEN improves QoL in patients with IF. The SF-36 score includes eight domains (physical functioning, role-physical, bodily pain, general health, vitality, social functioning, role-emotional, and mental health), all of which significantly improved. This finding is in line with those of other studies that suggested that HEN improves QoL (12, 35, 36). We speculate that the improvement of the patient's quality of life is mainly related to the improvement of skeletal muscle mass and function and the improvement of nutritional status, although further evidence appears necessary to fully investigate this issue.

We found that there were no significant increases in gastrointestinal, tube, or mechanical complications during HEN compared with the hospital treatment period. This finding suggests that HEN is safe as well as effective in patients with IF, similar to the findings of a previous study (14). Of note, HEN was not associated with any severe adverse effects.

Although guidelines recommend gastrostomy or jejunostomy as the first choice when nutritional support is necessary for more than 2–3 months (37), the nasogastric tube is easier to maintain in the home setting and some patients or caregivers can place the nasogastric tube by themselves. In the present study, the most common mode of administration for enteral nutrition was nasogastric feeding (42.2%). This can probably be explained by its wide use for patients with enteral nutrition of short duration, such as those with malignancies and inflammatory bowel diseases. The gastrostomy was more frequently applied in neuromuscular and gastroesophageal diseases (38). Jejunostomy was an exceptional alternative in our experience, as reported in other studies, this operation is only available to patients with severe and persistent gastroesophageal reflux or gastric dysmotility (38).

Our research results suggest that commercially manufactured standard types of enteral nutrition are the main type of formula used. This is due to commercial enteral nutrition convenience and ease of use that makes them the preferred choice among patients requiring formula. In clinical practice, patients with normal basic gastrointestinal function are recommended to take polymeric enteral feeding. However, some patients with altered GI function may require specialized formulas (i.e., severe hepatic diseases and malabsorption). Another noteworthy finding in our research is the low level of use of special formula enteral nutrition (i.e., fiber-rich, protein-rich, and energy-dense), accounting for only 7.8% of all patients using enteral nutrition. This may be made clear by the fact that standard enteral nutrition is well-tolerated by most patients. There is a lack of data on the benefits of special formula enteral nutrition in IF patients. Gravity feeding was rare in our study. This feeding route is unfit for IF patients because the precise flow cannot be achieved and the delivery target volume cannot be adjusted. Moreover, continuous enteral feeding is largely used in patients for both tolerance and acceptability, and flow regulator pumps were required for most patients receiving HEN (38).

Our study has certain limitations. First, our study had a limited sample size and relative heterogeneity of disease types. Nevertheless, the main focus of our observational, descriptive study was clinical outcomes within 6 months after discharge. Second, we did not have complete collection information on home parenteral nutrition use over the study period, as well as parenteral nutrition use time, amount, and proportion, all of which may have potential impact on prognosis.

In conclusion, the results of our study support the importance of HEN in malnourished patients with IF. HEN helps maintain body weight, body composition, PhA, and nutritional status with minimal safety concerns and has a positive impact on the quality of life.

## Data Availability Statement

The raw data supporting the conclusions of this article will be made available by the authors, without undue reservation.

## Ethics Statement

The studies involving human participants were reviewed and approved by the Research Ethics Committee of the Jinling Hospital. The patients/participants provided their written informed consent to participate in this study.

## Author Contributions

XW, XG, and JL equally contributed to the conception and design of the study. LZ and SL contributed to the design of the study. YZ contributed to the acquisition and analysis of the data. HL and DZ contributed to the analysis of the data. LZ contributed to the acquisition, analysis, and interpretation of the data. All authors drafted the manuscript, critically revised the manuscript, agreed to be fully accountable for ensuring the integrity and accuracy of the work, read, and approved the final manuscript.

## Conflict of Interest

The authors declare that the research was conducted in the absence of any commercial or financial relationships that could be construed as a potential conflict of interest.
