# Supplementary material for: Effect of Home Enteral Nutrition on Nutritional Status, Body Composition and Quality of Life in Patients With Malnourished Intestinal Failure
Source: Front Nutr. 2021 Jul 1;8:643907. doi: 10.3389/fnut.2021.643907 (PMC8281236; doi:10.3389/fnut.2021.643907)
Supplement: Supplementary file 1 [file Table_1.DOC]

**Table S1. Other clinical outcomes**

| **Work status** |  |
| --- | --- |
| Full-time | 24 (14.5) |
| Part-time | 106 (63.8) |
| Do not work | 36 (21.7) |
| **Survival** | 148 (89.2) |
| **Non-survival** | 18 (10.8) |
| **Diet** |  |
| Full oral diet | 60 (36.1) |
| Part oral diet | 86 (51.8) |
| Not oral diet | 20 (12.1) |
| **Energy and protein intake** |  |
| Received calories from EN (kcal/kg/day) | 18.5±4.6 |
| Received protein from EN (g/kg/day) | 0.86±0.35 |

Data are number of participants (%) or mean (SD).
