# Supplementary material for: Effect of Home Enteral Nutrition on Nutritional Status, Body Composition and Quality of Life in Patients With Malnourished Intestinal Failure
Source: Front Nutr. 2021 Jul 1;8:643907. doi: 10.3389/fnut.2021.643907 (PMC8281236; doi:10.3389/fnut.2021.643907)
Supplement: Supplementary file 2 [file Table_2.DOC]

**Table S2. Logistic regression analysis model assuming duration of HEN as exposure time and death as failure endpoint**

| Variable | Univariable | | Multivariable | |
| --- | --- | --- | --- | --- |
|  | HR (95% CI) | p-value | HR (95% CI) | p-value |
| **Age (years)**  ≥65/<65 | 4.917 (1.786–13.531) | 0.002 | 3.708 (1.200–11.453) | 0.023 |
| **Gender**  Male/Female | 0.754 (0.268–2.121) | 0.593 |  |  |
| **BMI (kg/m2)**  ≥16.5/<16.5 | 8.914 (1.979–40.150) | 0.004 | 9.558 (1.959–46.637) | 0.005 |
| **Disease type**  Tumor/Non–tumor | 3.229 (1.189–8.765) | 0.021 | 3.257 (1.006–10.546) | 0.049 |
| **Albumin (g/L)**  ≥35/<35 | 3.645 (1.275–10.418) | 0.016 |  |  |
| **[Prealbumin](../../../../E:/AppData/Local/youdao/dict/Application/8.5.1.0/resultui/html/index.html#/javascript:;) (mg/L)**  ≥200/<200 | 1.447 (0.508–4.412) | 0.489 |  |  |
| **IGF–1(µg/L)**  ≥100/<100 | 1.779 (0.644–4.911) | 0.267 |  |  |
| **Phase angle**  ≥3.74/<3.74 | 3.848 (1.341–11.043) | 0.012 |  |  |
